# The outcomes of laparoscopic omentum-preserving gastrectomy compared to open surgery with omentectomy in gastric cancer patients: a propensity score matched study of 249 UICC stage 0–IV gastric cancer patients

**DOI:** 10.1007/s00464-024-10835-6

**Published:** 2024-04-15

**Authors:** T. Jagric, G. Hladnik, R. Kolaric, I. Arpad, M. Horvat, S. Potrc

**Affiliations:** 1 Clinical Department for Abdominal and General Surgery, University Clinical Center Maribor, Ljubljanska 5, 2000 Maribor, Slovenia; 2 Department for Oncology, University Clinical Center Maribor, Ljubljanska 5, 2000 Maribor, Slovenia

**Keywords:** Omentum-preserving, Laparoscopic gastrectomy, D2 lymphadenectomy, Propensity score matching

## Abstract

**Background:**

We performed a propensity score matched study comparing patients’ short- and long-term results after laparoscopic omentum-preserving gastrectomy and open surgery with omentectomy with UICC stages 0–IV.

**Methods:**

Between 2015 and 2022, 311 patients with gastric cancer underwent surgery at the University Clinical Centre Maribor. Of these, 249 met the inclusion criteria and 198 were included in the study group after PSM.

**Results:**

Patients in both groups were well-balanced in demographic and pathological characteristics after PSM. There was no significant difference in the 5-year survival between groups (LAP: 62.2% vs. OPN: 54.4%; *p* = 0.950). The Cox regression model identified UICC stage and age as significant predictors for survival. In both groups, peritoneal dissemination was the most common site of recurrence. The multivariate analysis identified the UICC stage as a significant predictor for peritoneal recurrence, while omental preservation was not associated with a higher risk of peritoneal dissemination. Omentum preservation was not associated with more intestinal obstruction. Patients in the LAP group had significantly shorter hospital stays (LAP: 9(6) vs. OPN: 10(5); *p* = 0.009), less postoperative morbidity (LAP: 17% vs. OPN: 23.4%; *p* = 0.009), and significantly more extracted LNs per operation compared to open surgery (LAP: 31 ± 11 LNs vs. OPN: 25 ± 12 LNs; *p* = 0.002).

**Conclusion:**

Based on our results, we recommend the use of laparoscopic omentum-preserving gastrectomy in patients with early and advanced gastric cancer.

**Graphical abstract:**

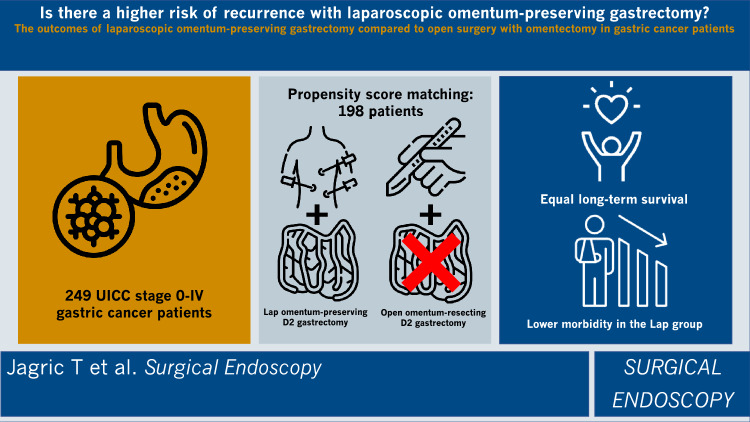

There is still an open debate regarding the need for a complete omentectomy with bursectomy in locally advanced gastric cancer [1–8]. While Kurokawa et al. have clearly shown in their study that bursectomy does not bring any survival benefits [1], the question of the potential role of omentectomy is far from being resolved [2–8]. No studies demonstrate the effectiveness of omentectomy in improving the local control of locally advanced diseases [2–8]. Moreover, the omentum has a potentially significant anti-inflammatory and tumor-protective role [1–8], it closes any anastomotic micro-leakages by adhesions to the neighboring structures [1–8], and it also has a substantial antitumor effect on free peritoneal tumor cells [2]. The partial preservation of the omentum greatly facilitates the laparoscopic gastrectomy and has therefore been liberally embraced by many laparoscopic surgeons and our center, where we adopted the omentum-preserving laparoscopic surgical technique used at Japan’s National Cancer Center Hospital East.

However, given that peritoneal dissemination is the most common type of recurrence after gastrectomy for gastric cancer, there is a substantial concern among surgeons that omentum preservation could potentially lead to more peritoneal recurrence. Furthermore, surgeons have feared that residual omentum after surgery could cause intestinal obstruction [3, 8]. Therefore, this study aimed to evaluate the impact of laparoscopic gastrectomy with omentum preservation on perioperative complications, peritoneal recurrence, postoperative intestinal obstruction occurrence, and long-term survival compared to open gastrectomy with omentectomy. We, therefore, performed a propensity score matched study comparing patients’ short- and long-term results after laparoscopic omentum-preserving gastrectomy and open surgery with omentectomy.

## Methods

This retrospective cohort study took place in a high-volume center for laparoscopic gastric cancer surgery (Department for General and Abdominal Surgery, University Clinical Center Maribor, Slovenia). Gastric cancer surgery at this hospital is highly standardized and performed by one dedicated surgical team who obtained their training at the National Cancer Center Hospital East in Japan, making the results more reliable. All patients referred to this team are scheduled for laparoscopic surgery. Patient data were pooled from the hospital database and the National Cancer Registry of Slovenia.

### Patients

All patients selected for this study had a complete preoperative work-up, including preoperative upper-gastrointestinal endoscopy. All patients had a verified stomach adenocarcinoma, and the exact tumor location was reported. In patients with early gastric cancer (EGC), tumor spotting was performed before the operation. A complete preoperative laboratory testing and chest X-ray were performed. In all patients, a chest and abdomen CT (computer tomography) scan was performed. A tumor board reviewed patients with complete preoperative staging before the operation. The eligibility criteria for perioperative treatment were as follows: UICC stage Ib gastric cancer or higher, age 18 years or older, ECOG 0 to 1, and adequate hepatic, renal, marrow, and cardiac function. Patients with a history of recent myocardial ischemia, uncontrolled angina, hypertension, cardiac arrhythmias, congestive heart failure, or other severe medical illnesses were taken under review by the tumor board. The regimen used for perioperative treatment was 5-FU–leucovorin–oxaliplatin–docetaxel (FLOT) four cycles pre- and four cycles post-operative. The precise doses for the regiment are provided in the as described elsewhere [9, 10]. The inclusion criteria for this study were adenocarcinoma diagnosed between 2015 and 2022, UICC tumor stages 0-IV, and complete follow-up. Patients with upfront unresectable gastric cancer, cancer in the stomach remnant, synchronous tumors, and patients lost during follow-up were excluded from the analysis. The demographic data, data regarding surgery, postoperative course, and pathological data were all stored in the hospital’s database. The follow-up data was collected from the national database. The follow-up and survival of the oncological patients in Slovenia is managed centrally in the National Oncological Database. This data is available for all clinicians treating cancer patients. The study was conducted according to the ethical directives of the Helsinki Declaration. Because of the retrospective study design, informed consent from the participants was waived by the Ethics committee.

### Surgery

Laparoscopic surgery was performed as follows: Briefly, upon trocar insertion, a thorough exploration of the abdominal cavity was performed. After the dissection of the right diaphragmatic pillar, the transaction of the phrenic-esophageal ligament, complete mobilization of the esophagus, fixation of the falciform ligament and the left liver lobe, we proceeded to the incision of the gastro-colic ligament starting 3 cm from the epiploic arcade. The mobilization of the esophagus was applied only in patients who received total gastrectomy, proximal subtotal gastrectomy, and the Ivor-Lewis procedure. The left gastroepiploic vessels were dissected at their take-off, taking special care not to injure the omental arteries. This was the point of gastric transection in subtotal gastrectomy. In total gastrectomy, we proceeded to dissection of the lymph node station No. 10. The dissection was continued up until the left diaphragmatic pillar was completely exposed. After completion of the lymphadenectomy of the splenic artery, the dissection was continued on the right side along the epiploic arcade. The gastroduodenal artery was exposed, and the distal part of the common hepatic artery, the right gastric artery, and the right gastroepiploic artery were identified. The anterior superior pancreaticoduodenal vein was the lowermost border of the No. 6 lymph node station dissection. The duodenum was transected with an EndoGIA 60 mm linear stapler with a purple cartridge (Medtronic, Minnesota, ZDA). The final step of the lymphadenectomy was the dissection of the lymph node stations 8, 11, 9 and 1. A 5–7 cm mini-laparotomy was made for specimen extraction. The reconstruction was mainly performed with a Roux-en-Y loop. An EndoGIA 60 mm linear stapled overlapping anastomosis was fashioned, and an air-leak test was performed [11, 12].

After surgery the patients were admitted for surveillance on the intensive care unit for two to three days. We discontinued the nasogastric tube and started clear fluids by mouth. The patients were allowed 100 ml clear fluids on day two, they were mobilized. The volume of fluids was slowly increased to day four, when they started with soft diet. The drains were removed on day 5 or when the secretion was below 200 ml per day. The patients were discharged on day 7.

Open surgery was performed similarly to laparoscopic surgery. The same lymph node stations were included in the lymphadenectomy as in laparoscopic surgery. In patients in the open group omentectomy was performed. This was done at the beginning of the dissection with the reflection of the omentum cranially and the incision of the gastrocolic ligament. The omentum was removed between the takeoff of the right and left gastroepiploic artery and resected en-block with the specimen.

### End-points, follow-up, and recurrence detection

The primary endpoint of the study was the 5-year overall survival. Overall survival was defined as the time from the operation until death from any cause. The secondary endpoints of the study were the morbidity according to Dindo-Claviene classification, the site of recurrence, the incidence of intestinal obstruction, and the length of hospital stay. Perioperative mortality was defined as death within 30 days of the procedure. Patients were regularly followed up after surgery. In the first two years after surgery, patients were followed every 4 months, then every 6 months until 5 years after surgery, then after once yearly. A complete physical exam was made, the tumor markers (CEA, Ca 19-9, Ca 72-4) were checked, and an abdominal ultrasound was performed at each control. In case of suspicion of tumor progression, a CT scan was performed. In patients with confirmed recurrence, the recurrence site was diagnosed with a CT scan, PET CT, operative exploration, or an autopsy.

### Statistical analysis

We defined six parameters associated with significant prognostic value after gastrectomy (tumor location, type of operation, UICC stage, ASA score, perioperative chemotherapy, and distant metastases). The PSM was performed with these variables to adjust for their confounding effects. The propensity matching was performed with the SPSS program, which uses a logistic model to estimate the propensity scores with the six variables as co-factors and automatically performs the nearest matching with a caliper of 0.1 in a ratio of 1:1.

Descriptive statistics were used to summarize patients’ demographic and clinical data. Continuous variables were expressed as mean ± SD or median with interquartile range (IQR) where appropriate. Categorical variables were expressed as absolute values with percentages. Continuous variables were compared with Student’s t-test and Mann–Whitney’s *U*-test where applicable. Variables above the threshold p-value of 0.1 were included for multivariate analysis. The binary logistic regression model was used to determine the significant predictor for peritoneal recurrence. The Cox regression model was used for primary analysis and included covariates with a p-value of more than 0.1 in univariate analysis. Treatment effect estimates were expressed as hazard ratios with a 95% confidence interval. Both multivariate logistic models used the backward stepwise conditional regression. Kaplan–Meier curves were constructed to determine time-to-event end-points. Differences in survivals between groups were determined with the Log-rank and Breslow tests. A *p*-value of > 0.05 was selected as the level of significance. All statistical analyses were performed on SPSS for Windows 10 v. 25 (IBM, USA).

## Results

### Patients

In total, 311 patients have been operated on for gastric cancer at our institution between 2015 and 2022. From these, 249 were eligible for our study (OPN; *n* = 143; LAP; *n* = 106). The included patients were divided into two groups: patients with open surgery and complete omentum resection and patients with laparoscopic gastrectomy and omentum preservation. After PSM, 202 patients were selected for the analysis (OPN; *n* = 101; LAP; *n* = 101). Of these patients, four patients were excluded because they were lost during follow-up, leaving 198 patients for the final analysis (LAP: *n* = 100; OPN: *n* = 98)(Patients’ demographics and tumor characteristics before and after PSM are presented in Table 1). Both groups were well balanced in the demographic and tumor characteristics after PSM.

**Table 1:** Patients and tumor characteristics

	Before PSM (*n* = 249)		*p*	After PSM (*n* = 198)		*p*
	Opn (143)	Lap (106)		Opn (98)	Lap (100)	
Age [years ± SD]	65 ± 11	69 ± 10	0.007*	66 ± 11	69 ± 11	0.065
Sex [n(%)]			0.053			0.166
M	104 (72.7)	66 (62.3)		70 (73.7)	64 (64)	
F	39 (27.3)	40 (37.7)		25 (26.3)	36 (36)	
Location [*n* (%)]			0.302			0.995
Pox 1/3	16 (11.3)	7 (6.6)		7 (7.4)	7 (7)	
Mid 1/3	60 (42.6)	46 (43.4)		42 (44.2)	45 (45)	
Dist 1/3	65 (46.1)	53 (50)		46 (48.4)	48 (48)	
ASA [*n* (%)]			0.01*			0.903
I	37 (26.1)	31 (29.2)		25 (26.3)	30 (30)	
II	77 (54.2)	53 (50)		51 (53.7)	51 (51)	
III	28 (19.7)	16 (15.1)		19 (20)	13 (13)	
IV	0 (0)	6 (5.7)		0 (0)	6 (6)	
Perioperative CT			0.094			0.106
No	51 (41.8)	57 (53.8)		39 (41.1)	51 (51)	
Yes	71 (58.2)	49 (46.2)		56 (58.9)	49 (49)	
Type of surgery [n (%)]			0.009*			0.266
GST	41 (28.7)	49 (46.2)		31 (32.6)	44 (44.0)	
GT	102 (71.3)	55 (51.9)		64 (67.4)	54 (54)	
GST prox	0 (0)	1 (0.9)		0 (0)	1 (1)	
Ivor-Lewis	0 (0)	1 (0.9)		0 (0)	1 (1)	
Surgery duration [min ± SD]	247.5 ± 65	277.5 ± 58	< 0.0001*	247.6 ± 65	276 ± 59	0.002*
D2 lymphadenectomy [*n* (%)]	113 (79.6)	80 (75.5)	0.227	72 (75.8)	78 (78)	0.547
No of LNs [number ± SD]	27.5 ± 14	31 ± 11	0.069	25 ± 12	31 ± 11	0.002*
R0 [*n* (%)]	140 (99.3)	100 (95.2)	0.171	94 (98.9)	95 (96)	0.503
DC [*n* (%)]			< 0.0001*			0.009*
0	91 (64.5)	86 (81.1)		68 (71.6)	83 (83)	
I	9 (6.4)	0 (0)		0 (0)	0 (0)	
II	29 (20.6)	5 (4.7)		17 (17.9)	5 (5)	
IIIa	4 (2.8)	0 (0)		3 (3.2)	0 (0)	
IIIb	4 (2.8)	11 (4.5)		4 (4.2)	10 (10)	
IV	3 (2.1)	3 (2.8)		2 (2.1)	1 (1)	
V	1 (0.7)	1 (0.4)		1 (1.1)	1 (1)	
30-day mortality [n (%)]	0 (0)	1 (0.9)	0.426	0 (0)	1 (1.1)	0.317
T [n (%)]			< 0.0001*			0.341
0	7 (5)	7 (6.6)		5 (5.3)	5 (5)	
1a	32 (22.7)	19 (17.9)		20 (21)	19 (19)	
1b	3 (2.1)	12 (11.3)		11 (11.6)	12 (12)	
2	12 (8.5)	15 (14.2)		8 (8.4)	15 (15)	
3	61 (43.3)	22 (20.8)		38 (40)	21 (21)	
4a	20 (14.2)	27 (25.5)		11 (11.6)	24 (24)	
4b	6 (4.3)	4 (3.8)		2 (2.1)	4 (4)	
N [n (%)]			0.357			0.575
0	62 (44)	59 (55.7)		43 (45.3)	56 (56.6)	
1	23 (16.3)	10 (9.4)		17 (17.9)	10 (10)	
2	26 (18.4)	16 (15.1)		19 (20)	14 (14)	
3a	13 (9.2)	10 (9.4)		8 (8.4)	10 (10)	
3b	17 (12.1)	11 (10.4)		8 (8.4)	10 (10)	
M [n(%)]	9 (6.4)	3 (2.8)	0.2	5 (5.3)	3 (3)	0.489
UICC[n(%)]			0.749			0.557
0	5 (4.4)	7 (6.6)		3 (3.2)	5 (5)	
Ia	29 (25.7)	29 (27.4)		27 (28.5)	29 (29)	
Ib	8 (7.1)	10 (9.4)		8 (8.4)	10 (10)	
IIa	13 (11.5)	12 (11.3)		12 (12.6)	12 (12)	
IIb	15 (13.3)	11 (10.4)		11 (11.6)	10 (10)	
IIIa	19 (16.8)	15 (14.2)		16 (16.8)	13 (13)	
IIIb	10 (8.8)	14 (13.2)		9 (9.5)	14 (13)	
IIIc	7 (6.2)	6 (5.7)		6 (6.3)	6 (6)	
IV	7 (6.2)	2 (1.9)		3 (3.2)	2 (2)	
Hospital stay [days(IRQ)]	10 (5)	9 (7)	0.013*	10 (5)	9 (6)	0.009*
Recurrence site [n(%)]			0.016*			0.594
No recurrence	92 (64.3)	85 (80.2)		65 (68.4)	78 (38.9)	
Peritoneum	38 (26.6)	15 (14.2)		22 (23.2)	16 (16)	
Liver	7 (4.9)	0 (0)		4 (4.2)	0 (0)	
Anastomosis	1 (0.7)	1 (0.9)		0 (0)	1 (1)	
Lymph nodes	2 (1.4)	1 (0.9)		1 (1.1)	1 (1)	
Abdominal wall	0 (0)	1 (0.9)		0 (0)	1 (1)	
Lung	1 (0.7)	2 (1.9)		1 (1.1)	3 (3)	
Bones	2 (1.4)	1 (0.9)		2 (2.1)	1 (1)	
Intestinal obstruction [*n*(%)]			0.083			0.949
No	134 (93.7)	104 (98.1)		92 (96.8)	97 (97)	
Yes	9 (6.3)	2 (1.9)		3 (3.2)	3 (3)	

*Opn* open gastrectomy with complete omentectomy, *Lap* Laparoscopic gastrectomy with omentum preservation; *M* Male; *F* Female; *Prox 1/3* Proximal third gastric cancer; *Mid 1/3* middle third gastric cancer; *Dist 1/3* Distal third gastric cancer; *Perioperative CT* perioperative chemotherapy; *GST* subtotal gastrectomy; *GT* total Gastrectomy; *GST prox* proximal subtotal gastrectomy; *No of LNs* number of lymphnodes; *DC* dindo-claviene

### Perioperative results

In both groups, the total gastrectomy was the most prevalent procedure (OPN: 67.4% vs LAP: 54%; *p* = 0.266). Open surgery with omentectomy was on average 30 min faster compared to laparoscopic omentum-preserving gastrectomy (247.6 ± 65 in OPN vs 276 ± 59 in LAP; *p* = 0.002). D2 lymphadenectomy was performed in the same proportion of patients in both groups (LAP: 78% vs. OPN: 75.8%; *p* = 0.547). Meanwhile, the lymph node yield was significantly higher in the LAP group (31 ± 11 LNs vs. 25 ± 12 LNs; *p* = 0.002). The conversion rate in the Lap group was 16%. The reasons for conversion are listed in Table 2. In both groups, R0 resection was achieved in the same proportion (LAP: 96% vs. OPN: 98.9%; *p* = 0.503). Most of the patients had advanced gastric cancer (pT2 or higher) (LAP: 64% vs. OPN: 62.1%; *p* = 0.341). Patients with pathological stage T0 were included in both groups. These patients were diagnosed with gastric adenocarcinoma on preoperative biopsy and were found to have a complete tumor regression after chemotherapy. All preoperative biopsies were revised to confirm the diagnosis.

**Table 2:** Conversions to open surgery in the Lap group

	Lap group after PSM (*n* = 100)
Conversion [n(%)]	16 (16)
Reason for conversion [n(%)]* (*n* = 16)	
Anastomosis	3 (18.8)
Adhesions	1 (6.3)
Bleeding	8 (50)
Tumor infiltration	4 (25)

Patients in the LAP group had significantly less morbidity compared to OPN group (LAP: 17% vs. OPN: 23.4%; *p* = 0.009). There was no significant difference in the 30-day morbidity between groups (LAP: 1.1% vs. OPN: 0%; *p* = 0.317). Patients in the LAP group had a significantly shorter hospital stay compared to OPN group (9(6) days vs. 10(5) days; *p* = 0.009). Perioperative chemotherapy was administered in a similar proportion of patients in both groups (LAP: 49% vs. OPN 58.9%; *p* = 0.106).

### Recurrence and intestinal obstruction

In both groups, the most common site of recurrence was the peritoneum (LAP: 16% vs. OPN: 23.2%; *p* = 0.594). Patients in the LAP group had less frequent peritoneal recurrence compared to OPN group and more frequent recurrence in the lungs (LAP: 3% vs. OPN: 1.1%), while liver metastases were more common in the OPN group (LAP: 0% vs. OPN: 4.2%). All former differences did not reach the level of significance. In both groups, recurrence in local or distal LNs was exceedingly rare (LAP: 1% vs. OPN: 1.1%; *p* = 0.594). Also, there was no significant difference in the occurrence of intestinal obstruction. Intestinal obstruction was observed in 3.2% in OPN and in 3% in LAP group (*p* = 0.949).

A logistic regression model was performed to determine the most significant predictors for peritoneal recurrence. From the included variables, only UICC stage (OR 0.373; 95%CI 0.216–0.609; *p* < 0.0001) was determined to be a significant predictor for peritoneal recurrence.

### Survival analysis

**Fig. 1:**
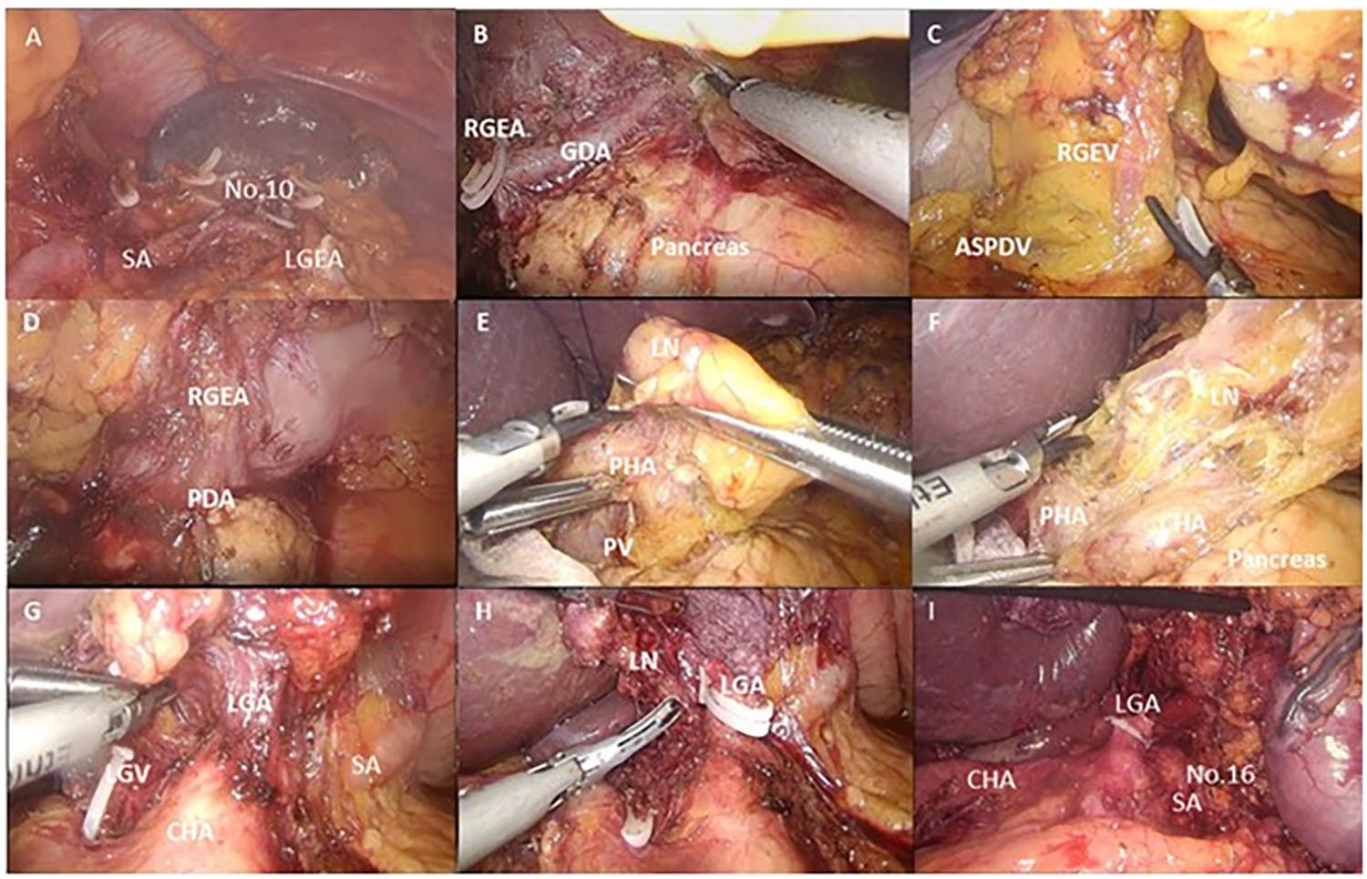
Laparoscopic lymph node dissection in total gastrectomy. *SA* splenic artery; *LGEA* left gastroepiploic artery; *GDA* gastroduodenal artery; *RGEA* right gastroepiploic artery; *ASPDV* anterior superior pancreaticoduodenal vein; *RGEV* right gastroepiploic vein; *RGEA* right gastroepiploic artery; *PDA* pancreaticoduodenal artery; *PV* portal vein; *PHA* proper hepatic artery; *LN* lymph nodes; *F* PHA: proper hepatic artery; *CHA* common hepatic artery; *No.10* lymph node station No.10; *N0.16* lymph node station No.16; *LGV* left gastric vein; *LGA* left gastric artery; *LA* lienal artery

**Fig. 2:**
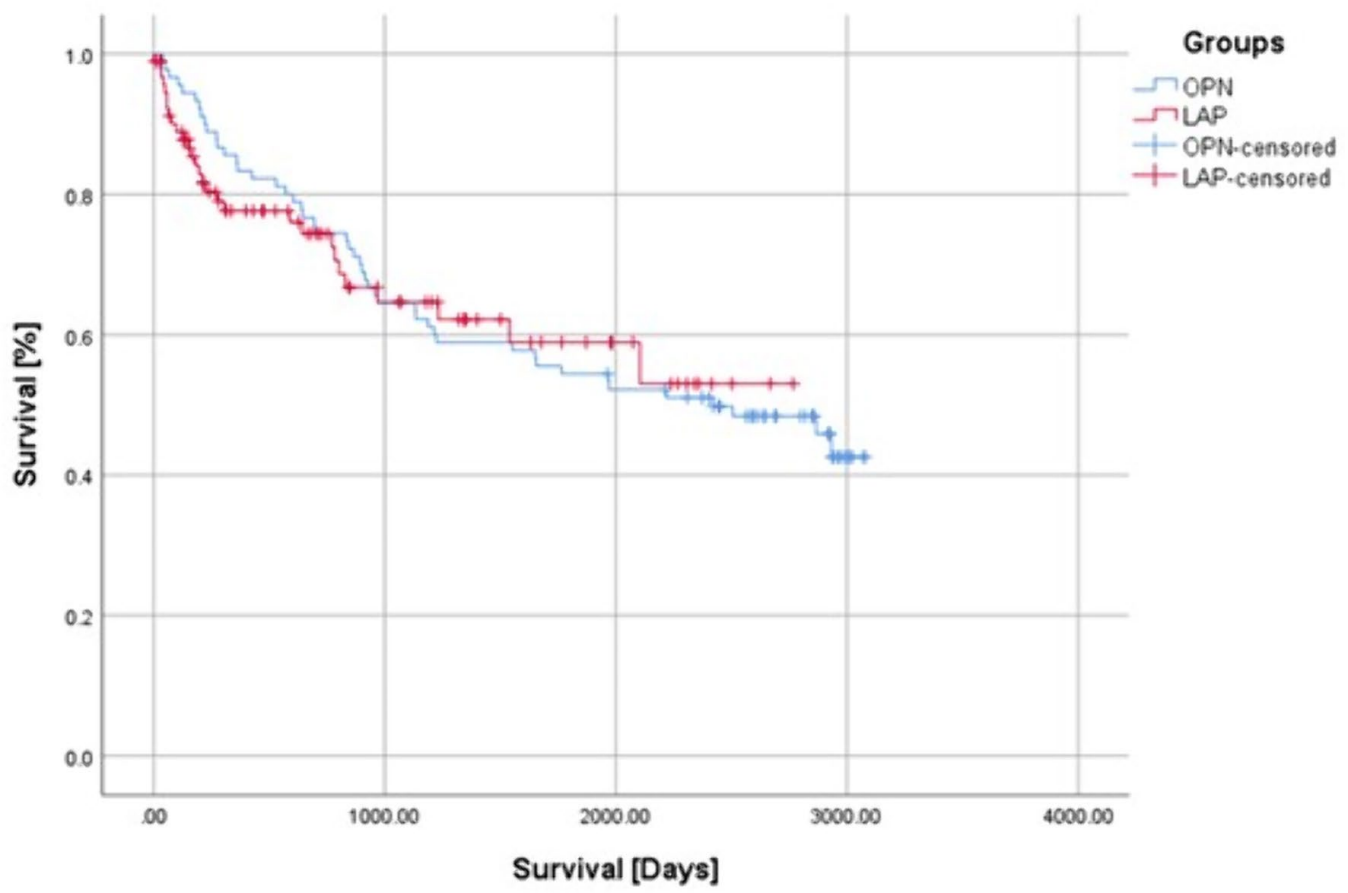
Overall survival after laparoscopic gastrectomy with omentum preservation and open gastrectomy with omentectomy

The median follow-up period was 3.6 (5.8) years. There was no significant difference in the long-term survival between LAP and OPN groups (*p* = 0.950). The 5-year overall survival was 62.2% in LAP and 54.4% in OPN group (Fig. 1). From the included variables, the Cox proportional hazard model determined UICC (HR: 2.230; 95%CI 1.734–2.867; *p* < 0.0001) and age (HR: 1.057; 95%CI 1.030–1.086; p < 0.0001) to be significantly associated with long-term survival (Fig. 2).

## Discussion

Omentectomy has never been shown to provide a survival benefit in gastric cancer patients [1–8]. Despite that, it is still recommended as the standard treatment for advanced gastric cancer [13]. Omentum preservation dramatically facilitates the operation and reduces intraoperative and postoperative complications in laparoscopy. Meanwhile, some concerns have been raised that omentum preservation could lead to more peritoneal recurrence in patients with advanced gastric cancer. In the present paper, we confirmed that omentum resection did not provide any survival benefit compared to laparoscopic gastrectomy with omentum preservation.

Our study showed no differences in the 5-year survivals between patients after laparoscopic omentum-preserving gastrectomy and open surgery with omentectomy. In the multivariate analysis, only the UICC stage and age were determined to have significantly impacted survival in our patient group, while the complete omentectomy did not have any significant impact on the overall survival. Similar results were obtained by other retrospective studies [3–7]. These studies are prone to selection bias due to their retrospective nature. We adjusted for the most significant factors influencing survival to avoid such a selection bias. Our patients were balanced in regard to UICC stage distribution, age, ASA grade, tumor location, type of operation, and were all operated on in the same period. An important factor to consider in patient selection is chemotherapy. Perioperative treatment is the standard for patients with stages Ib or higher in Europe [13]. The FLOT4 study demonstrated that patients receiving chemotherapy have a significant survival advantage [14]. In the study conducted by Hasegawa et al., there was a significant difference in the proportion of patients receiving adjuvant treatment, with patients in the omentum-preserving group receiving adjuvant treatment in a greater proportion [5]. This might have influenced the comparability of the results. To adjust for this factor in our study, perioperative treatment was used as a co-factor for PSM, making our patient groups well-balanced.

Omentectomy has the advantage of theoretically removing microscopic tumor seeds and preventing the spread of malignancy [5]. If omentectomy prevented peritoneal recurrence, we would expect significant differences in the modes of recurrence between LAP and OPN groups. Our study showed no difference in the recurrence sites between groups. Peritoneal recurrence was the most common type of recurrence in both groups, as reported by other authors [5–7]. The logistic regression model confirmed that only the UICC stage was significantly associated with peritoneal recurrence and that laparoscopic gastrectomy with omental preservation did not lead to a higher risk for peritoneal recurrence.

The patients in LAP group had lower morbidity rates compared to OPN group. Complete omentectomy has been shown to be related to a higher incidence of complications [3–8]. Our results support these findings. Omentum preservation facilitates the laparoscopic resection, shortens the time of the surgery, and produces less blood loss [1–8]. It also has an important anti-inflammatory role and can adhere to the site of potential leaks from the anastomosis [1–8]. Our morbidity rates compare favorably to recent RCTs where patients received a laparoscopic total gastrectomy in a similar proportion as in our study [15–17]. The mortality rates in both groups were 1% and comparable to previous reports on laparoscopic gastrectomies from Eastern RCTs [18–22]. An important late morbidity to consider is intestinal obstruction. Theoretically, the preserved omentum could cause intestinal adhesions and obstruction. Our results confirmed that omentum preservation did not lead to more intestinal obstructions. Finally, patients in the LAP group had significantly shorter hospital stays compared to OPN group. The JCOG0912 and KLASS01 trials have demonstrated superior postoperative recovery in terms of shorter hospital stays after laparoscopy compared to open surgery [18, 19]. This was also evident in the present study.

In our study, a D2 dissection was performed in 75% to 78% of cases. Meanwhile, patients in the LAP group had a significantly higher number of extracted lymph nodes per operation compared to OPN group. Thus, laparoscopy and omental preservation did not lead to an inferior quality of lymph node dissection. On the contrary, we feel that laparoscopy might be associated with a more precise lymph node dissection due to magnification and better anatomical exposure than open surgery. A similar result was published in a recent meta-analysis by Liao et al., where an insignificantly higher lymph node was obtained in laparoscopically operated patients [23].

This is the first Western study comparing the oncological feasibility of laparoscopic omentum-preserving gastrectomy compared to open surgery with omental resection. We could only find one study from a Western center with results of omentum-sparing laparoscopic surgery [24]. Therein, Olmi et al. presented their ten-year experience of laparoscopic omentum-preserving gastrectomy. Nevertheless, they did not compare omentum-preserving gastrectomy to omentum-resecting gastrectomy making the results difficult to interpret in the light of oncological safety. All other reports on omentum-sparing gastrectomy are from Eastern centers where the incidence of gastric cancer, stage distribution, and patient demographics differ from the West [3–8].

This study has several limitations. It was performed on a relatively small patient sample. Nevertheless, the necessary number of participants for a non-inferiority study with 80% power, a standard deviation of 5%, and a margin of error of 5% is 72 patients in each arm. In our paper, more than 90 patients remained in each arm after PSM. Another limitation was the mixed pathological tumor stage population. Most of the patients had advanced gastric cancer, making it safe to assume that our results can be generalized to patients with advanced gastric cancer. Lastly, the present paper is a retrospective study. The PSM could account for some of the selection bias. However, many subtle factors that have not been accounted for may have been missed. Therefore, performing a multi-institutional randomized controlled trial might be of significant value. Despite all these limitations, we believe that single-center studies provide a valuable source of information regarding the feasibility of laparoscopic omentum-preserving gastric cancer surgery in the West, where large-scale studies are much more challenging to produce.

The results from this single high-volume center support that the laparoscopic omentum-preserving gastrectomy was oncologically equivalent to open surgery with omentectomy, with less perioperative morbidity and faster postoperative recovery and discharge. Based on our results, we recommend the use of laparoscopic omentum-preserving gastrectomy in patients with early and advanced gastric cancer.

## References

[1] Kurokawa Y, Doki Y, Mizusawa J, Terashima M, Katai H (2018). Bursectomy versus omentectomy alone for resectable gastric cancer (JCOG1001): a phase 3, open-label, randomised controlled trial. Lancet.

[2] Olmi S, Uccelli M, Oldani A, Cesana G, Ciccarese F, Giorgi R (2020). Laparoscopic surgery of gastric cancer with D2 lymphadenectomy and omentum preservation: Our 10 years experience. J Laparoendosc Adv Surg Tech A.

[3] Li Z, Song M, Zhou Y, Jiang H, Xu L, Hu Z, Liu Y, Jiang Y, Li X (2021). Efficacy of omentum-preserving gastrectomy for patients with gastric cancer: a systematic review and meta-analysis. Front Oncol.

[4] Kim DJ, Lee JH, Kim W (2014). A comparison of total versus partial omentectomy for advanced gastric cancer in laparoscopic gastrectomy. World J Surg Oncol.

[5] Hasegawa S, Kunisaki C, Ono H, Oshima T, Fujii S, Taguri M (2013). Omentum-preserving gastrectomy for advanced gastric cancer: a propensity-matched retrospective cohort study. Gastric Cancer.

[6] Sakimura Y, Inaki N, Tsuji T, Kadoya S, Bando H (2020). Long-term outcomes of omentum-preserving versus resecting gastrectomy for locally advanced gastric cancer with propensity score analysis. Sci Rep.

[7] Ri M, Nunobe S, Honda M, Akimoto E, Kinoshita T (2020). Gastrectomy with or without omentectomy for cT3-4 gastric cancer: a multicentre cohort study. Br J Surg.

[8] Murakami H, Yamada T, Taguri M, Hasegawa S, Yamanaka T, Rino Y (2021). Short-term outcomes from a randomized screening phase II. Non-inferiority trial comparing omentectomy and omentum preservation for locally advanced gastric cancer: the TOP-G Trial. World J Surg.

[9] Jagric T, Ilijevec B, Velenik V, Ocvirk J, Potrc S (2019). Impact of perioperative treatment on survival of resectable gastric cancer patients after D2 lymphadenectomy: a single European center propensity score matching analysis. Radiol Oncol.

[10] Lordick F, Carneiro F, Cascinu S, Fleitas T, Haustermans K, Piessen G, Vogel A, Smyth EC (2022). On behalf of the ESMO Guidelines Committee. Gastric cancer: ESMO clinical practice guideline for diagnosis, treatment and follow-up. Ann Oncol.

[11] Uyama I, Sugioka A, Matsui H, Fujita J, Komori Y, Hasumi A (2000). Laparoscopic D2 lymph node dissection for advanced gastric cancer located in the middle of the lower third portion of the stomach. Gastric Cancer.

[12] Kayana S, Haruta S, Kawamura Y, Yoshimura F, Inaba K, Hiramatsu Y, Ishida Y, Taniguchi K, Isogaki J, Uyama I (2011). Video: distinctive laparoscopy technique for supra pancreatic lymph node dissection: medial approach for laparoscopic gastric cancer surgery. Surg Endosc.

[13] Lordick F, Carneiro F, Cascinu S, Fleitas T, Haustermans K, Piessen G (2022). Gastric cancer: ESMO clinical practice guideline for diagnosis, treatment and follow-up. Ann Oncol.

[14] Al-Batran SE, Hofheinz RD, Pauligk C, Kopp HG, Haag GM, Luley KB (2016). Histopathological regression after neoadjuvant docetaxel, oxaliplatin, fluorouracil, and leucovorin versus epirubicin, cisplatin, and fluorouracil or capecitabine in patients with resectable gastric or gastro-oesophageal junction adenocarcinoma (FLOT4-AIO): results from the phase 2 part of a multicentre, open-label, randomised phase 2/3 trial. The Lancet.

[15] van der Wielen N, Straatman J, Daams F, Rosati R, Parise P (2021). Open versus minimally invasive total gastrectomy after neoadjuvant chemotherapy: results of a European randomized trial. Gastric Cancer.

[16] van der Veen A, Brenkman HJF, Seesing MFJ, Haverkamp L, Luyer MDP (2021). Laparoscopic versus open gastrectomy for gastric cancer (LOGICA): a multicenter randomized clinical trial. J Clin Oncol.

[17] Yang HK, Hyung WJ, Han SU, Lee YJ, Park JM (2020). Comparison of surgical outcomes among diferent methods of esophagojejunostomy in laparoscopic total gastrectomy for clinical stage I proximal gastric cancer: results of a single-arm multicenter phase II clinical trial in Korea, KLASS 03. Surg Endosc.

[18] Katai H, Mizusawa J, Katayama H, Takagi M, Yoshikawa T (2017). Short-term surgical outcomes from a phase III study of laparoscopy-assisted versus open distal gastrectomy with nodal dissection for clinical stage IA/IB gastric cancer: Japan Clinical Oncology Group Study JCOG0912. Gastric Cancer.

[19] Kim W, Kim HK, Han SU, Kim MC, Hyung WJ (2016). Decreased morbidity of laparoscopic distal gastrectomy compared with open Distal gastrectomy for stage i gastric cancer: short-term outcomes from a multicenter randomized controlled trial (KLASS-01). Ann Surg.

[20] Nam BH, Kim YW, Reim D, Eom BW, Yu WS (2013). Laparoscopy assisted versus open distal gastrectomy with D2 lymph node dissection for advanced gastric cancer: design and rationale of a phase II randomized controlled multicenter trial (COACT 1001). J Gastric Cancer.

[21] Inaki N, Etoh T, Ohyama T, Uchiyama K, Katada N (2015). A multi-institutional, prospective, phase II feasibility study of laparoscopy-assisted distal gastrectomy with D2 lymph node dissection for locally advanced gastric cancer (JLSSG0901). World J Surg.

[22] Hu Y, Huang C, Sun Y, Su X, Cao H, Hu J (2016). Morbidity and mortality of laparoscopic versus open D2 distal gastrectomy for advanced gastric cancer: a randomized controlled trial. J Clin Oncol.

[23] Liao XL, Liang XW, Pang HY, Yang K, Chen XZ (2021). Safety and efficacy of laparoscopic versus open gastrectomy in patients with advanced gastric cancer following neoadjuvant chemotherapy: a meta-analysis. Front Oncol.

[24] Olmi S, Uccelli M, Oldani A, Cesana G, Ciccarese F, Giorgi R (2020). Laparoscopic surgery of gastric cancer with D2 lymphadenectomy and omentum preservation: Our 10 years experience. J Laparoendosc Adv Surg Tech.

